# Hypo-endemic onchocerciasis hotspots: defining areas of high risk through micro-mapping and environmental delineation

**DOI:** 10.1186/s40249-015-0069-6

**Published:** 2015-08-16

**Authors:** Louise A. Kelly-Hope, Thomas R. Unnasch, Michelle C. Stanton, David H. Molyneux

**Affiliations:** Liverpool School of Tropical Medicine, Pembroke Place, Liverpool, L3 5QA UK; University of South Florida, Tampa, Florida USA

**Keywords:** Onchocerciasis, River blindness, *Loa loa*, Loiasis, Severe adverse events, Mapping, Elimination, Environment, Africa

## Abstract

**Background:**

Onchocerciasis (river blindness) caused by the parasite *Onchocercavolvulus* and transmitted by riverine *Simulium* spp. (Black flies) is targeted for elimination in Africa. This is a significant change in strategy from the ‘control’ of meso- and hyper-endemic areas through mass drug administration (MDA) with Mectizan® (ivermectin), to the ‘elimination’ in all endemic areas where a range of interventions may be required. The most significant challenges of elimination in low transmission or hypo-endemic areas are two-fold. First, there are vast remote areas where the focality of low transmission is relatively undefined. Second, the treatment with ivermectin increases the risk of serious adverse events (SAEs) in individuals with high parasitaemias of *Loa loa*, a filarial parasite widespread in Central and West Africa, which causes Tropical eye worm and transmitted by *Chrysops* spp. (Deer flies).

**Discussion:**

We therefore propose novel mapping approaches using remote sensing satellite and modelled environmental data to be used in combination with rapid field surveys to help resolve the problems of targeting the expansion of onchocerciasis elimination activities in *L. loa* co-endemic areas. First, we demonstrate that micro-stratification overlap mapping (MOM) of available onchocerciasis and loiasis prevalence maps can be used to identify 12 key high risk areas, where low *O. volvulus*and high *L. loa* transmission overlap, which we define as “hypo-endemic hotspots”. Second we show that integrated micro-mapping of prevalence data, and the use of environmental data to delineate riverine and forest risk factors associated with *Simulium* spp. and *Chrysops* spp. vector habitats can further help to define target intervention areas i.e. secondary hotspots within hotspots, to help avoid the risk of SAEs.

**Summary:**

These mapping examples demonstrate the value of bringing prevalence, entomological and ecological information together to develop maps for planned implementation and targeted strategies. This is critical as better mapping may the reduce costs and lower the *L. loa* associated risks, especially if there are extensive areas of low endemicity that may require treatment with ivermectin or alternative strategies. Novel cost-effective approaches are necessary if elimination of *O.volvulus* transmission in Africa is to be achieved in an efficient and safe way by the goal of 2025.

**Electronic supplementary material:**

The online version of this article (doi:10.1186/s40249-015-0069-6) contains supplementary material, which is available to authorized users.

## Multilingual abstracts

Please see Additional file 1 for translations of the abstract into the six official working languages of the United Nations.

## Background

Onchocerciasis (river blindness) is a neglected tropical disease (NTD), caused by the parasite *Onchocerca volvulus* and transmitted by riverine vector of *Simulium* spp.(Blackflies) [1, 2]. The highest burden of onchocerciasis occurs in Africa, and over the past two decades the African Programme for Onchocerciasis Control (APOC) has focussed its efforts on the control of high transmission meso- and hyper-endemic areas through mass drug administration (MDA) with the drug Mectizan® (ivermectin), based on the community-directed treatment with ivermectin (CDTi) platform [3–5]. The CDTi approach has largely been effective and sustainable, and in 2010 a momentous decision was made to expand the programme to include all endemic areas and aim for the elimination of onchocerciasis by the year 2025 [6, 7].

The decision to eliminate *O.volvulus* transmission in Africa poses significant logistic and resource challenges to country programmes [7, 8]. However, most importantly before any interventions can be scaled up in the new low transmission or hypo-endemic target areas, it will be essential to define the distribution and drivers of transmission as they were not examined in detail following the initial rapid epidemiological mapping activities [9, 10], and the epidemiology may differ significantly to high transmission areas. Further, the geographical areasare vast and remote, and many communities in these new target areas are drug naïve to ivermectin. The treatment with ivermectin also poses risks of serious adverse events (SAEs) in individuals with high *L. loa* parasitaemias [11, 12]; therefore,it will be critical to clearly map the co-distribution with the filarial parasite *Loa loa*, which is transmitted by *Chrysops* spp. (Deer flies) [13].

Thus, onchocerciasis elimination in highly endemic *L. loa* areas will require alternative treatment strategies [8], which may be adapted to the infection in the human population or the local ecology of the *Simulium* spp. vector. To begin to address these challenges, we put forward the idea that additional innovative mapping methods using remote sensing satellite technologies and modelled environmental data are essential to help identify where the low onchocerciasis transmission overlaps with high risk *L. loa* areas, which we describe as *‘hypo-endemic hotspots’.* We also hypothesize that within these hotspot zones it is possible to further delineate target areas using environmental determinants of the specific vector habitats. Here, we present the case for- and provide examples of- two new mapping approaches which can be used in combination with the standard rapid epidemiological methods, to define the high risk hypo-endemic communities requiring alternative treatment strategies. This will reduce the time and increase the efficiency in which they are identified, and help to reduce the extent and related costs of additional field surveys.

## Discussion

### Onchocerciasis elimination in context

The decision to progress towards the elimination of *O.volvulus* in Africa matches the objectives of the Onchocerciasis Elimination Programme of the Americas (OEPA), which successfully used a strategy of twice yearly treatment of ivermectin in onchocerciasis endemic communities, with two countries verified free of transmission, and three countries under surveillance with dossiers detailing the data evidence for certification submitted [6, 14–18]. In Africa, the decision for elimination was based on studies in previously highly endemic areas of Senegal and Mali in the basins of the Gambia, Bakoye and Faleme rivers, where it was demonstrated that between 15 to 17 years of twice annual or annual ivermectin treatment reduced the prevalence to zero in most communities. There was also a parallel reduction in the transmission as measured by pool screening of *Simulium* spp., where the estimated upper bound of the 95 % confidence of the prevalence of flies carrying infective larvae was less than 1/1000 parous flies collected [19]. Similar results in Kaduna State, Nigeria following 15 years of annual CDTi confirmed that *O. volvulus* prevalence had declined to zero in two previously highly endemic foci [19–21].

Initially, the objective of APOC was to sustain the delivery of ivermectin via CDTi project areas of meso- and hyper-endemicity defined by two rapid mapping methods, Rapid Epidemiological Assessment (REA) and Rapid Epidemiological Mapping of Onchocerciasis (REMO) [9, 10], using nodule palpation in 30–50 adult males over the age of 20 years per village [2]. These rapid epidemiological mapping methods use geographic attributes to determine areas likely to be suitable for *Simulium* spp. breeding. This approach is rooted in the fact that the vectors of *O. volvulus* have highly specific riverine breeding site requirements, and therefore with the aid of topographical maps, it was possible to make a choice of representative villages most likely to be seriously affected by onchocerciasis located ≤10 km from the riverine breeding sites [9, 10]. APOC also supported vector control in certain settings where *Simulium* spp. populations were isolated and there was limited risk of reinvasion. (e.g. foci in Uganda, Tanzania and on the island of Bioko, Equatorial Guinea) [4, 22, 23].

The decision to target the elimination of onchocerciasis in Africa through MDA has wide logistic, financial, human resource and policy implications. APOC projects were initially confined to meso- and hyper-endemic areas, but now extend to include hypo-endemic areas, referred to as ‘low transmission zones’, where the nodule prevalence was found to be less the 20 % in REMO surveys, which correlate with <35 % microfilaria (Mf) in the skin [3, 24]. Communities in these low transmission zones were not previously treated, as they were considered of less public health importance. Therefore, extensive areas of lower endemicity outside existing treatment zones may now require treatment with ivermectin. However the treatment numbers, locations and related critical cut-offs of who and where to treat and not-to-treat are not well defined. In 2011,an estimated 77.3 million people living in 2.8 million km^2^ had 5–19.9 % nodule prevalence, and another 97.6 million in 3 million km^2^had <5 % nodule prevalence [3]. These estimates were based on 2011 population estimates, and it is possible prevalence figures pertain to an era before the start of control (before 2011). It is also possible that they have changed over the past 5 years, and either increased with population growth or movement, or decreased with the potential impact of other large scale MDA programmes such as lymphatic filariasis (LF), which also distributes ivermectin [25]. Understanding the population dynamics and overlaps with other NTD programmatic activities will be particularly important in large highly populated countries such as Nigeria and the Democratic Republic of Congo (DRC) [26, 27].

Rapid delineation of these low transmission zones to determine their geographical limits, and the numbers requiring treatment is now critical for the expansion of the programme and if the goal of elimination is to be achieved. This is particularly pertinent as the number of people and geographical area to cover with interventions (standard or alternative), is more than double the size of meso- and hyper-endemic areas, and the epidemiology less defined. Further, it will be important to consider the complexities of *L. loa* co-endemicity where alternative strategies will be required [8, 28]. Previous mapping methods of REA and REMO used to define CDTi project areas may not be practical to use for the low transmission zones, given the long time in the field they take to conduct, and the need to access such large populations, in extensive and remote geographical areas. Therefore, alternative innovative approaches are necessary to increase the speed and efficiency in which to identify hypo-endemic target areas, which need to be mapped and treated.

### The problem with *Loa loa*

The wide distribution of *L. loa* filariasis through Central and West Africa has and continues to pose a major obstacle for onchocerciasis elimination because the standard strategy of community wide treatment with ivermectin, cannot be used due to the risk of SAEs and possible deathof individuals with high *L. loa* Mf loads. The risk of SAEs is high when the Mf loads exceed 8000 Mf/ml, and the reaction is more severe if the load exceeds 30,000 Mf/ml [29–31]. APOC began to address this problem over a decade ago by implementing a rapid mapping method, Rapid Assessment Procedure for Loiasis (RAPLOA), which is based on eye worm history and assesses the proportion of infected people in a community [13]. The large-scale RAPLOA field surveys helped produce endemicity risk maps for programmatic use. RAPLOA prevalence >40 % broadly correlates with >20 % Mf prevalence, and defines the high risk areas. However RAPLOA is limited in that the proportion of the population with Mf loads over 8,000 Mf/ml and 30,000 Mf/ml is not well defined across large areas, and these can vary considerably across different human and geographical landscapes [32–34]. This uncertainty in risk within populations has implications for the choice of alternative strategies, which may include different drug regimens and/or targeted vector control.

### New mapping approaches

We have previously developed two mapping strategies to address the problems associated with *L. loa* co-endemicity. The first, the micro-stratification overlap mapping (MOM) strategy, has been used to determine the levels at which the implementation of LF programmes is most efficiently deployed in *L. loa*co- endemic areas, using examples from the DRC, Nigeria and South Sudan [26, 27, 35]. The MOM strategy has also been used to broadly define environmental characteristics of *L. loa* in Central Africa around the Congo River Basin [36], and to highlight the zoogeography of filarial infections [37], building on the initial use of remotely sensed imagery associated with *L. loa* prevalence data [38]. The second, the integrated micro-mapping strategy, has been used to inform mapping and alternative treatment strategies for LF elimination in *L. loa* co-endemic areas [39], and also to overlap different *O. volvulus* and *L. loa* transmission zones at a micro-epidemiologic scale in order to highlight the prevalence distribution of SAEs in DRC [12].

Here, we expand on these strategies, and hypothesise that by using these additional mapping methods, we can first broadly identify where the main ‘*hypo-endemic hotspots’*are located. Second, we can delineate the high risk and treatment target areas within each hotspot using the environmental determinants of the riverine breeding sites of *Simulium* spp*.* [40, 41], and the forest canopy habits of *Chrysops* spp. where biting rates are likely to be highest and hence transmission will be concentrated [42–44]. Both methods use geographical information systems (GIS) software to examine high resolution remote sensing satellite imagery and modelled environmental data. Compared with field surveys that could take months to years to complete with high costs associated with personnel and transport, these mapping methods require the specialised knowledge and skills of a few GIS, remote sensing and environmental experts, which could complete the related work within weeks to months.

### Hypo-endemic hotspot case studies

**Fig. 1:**
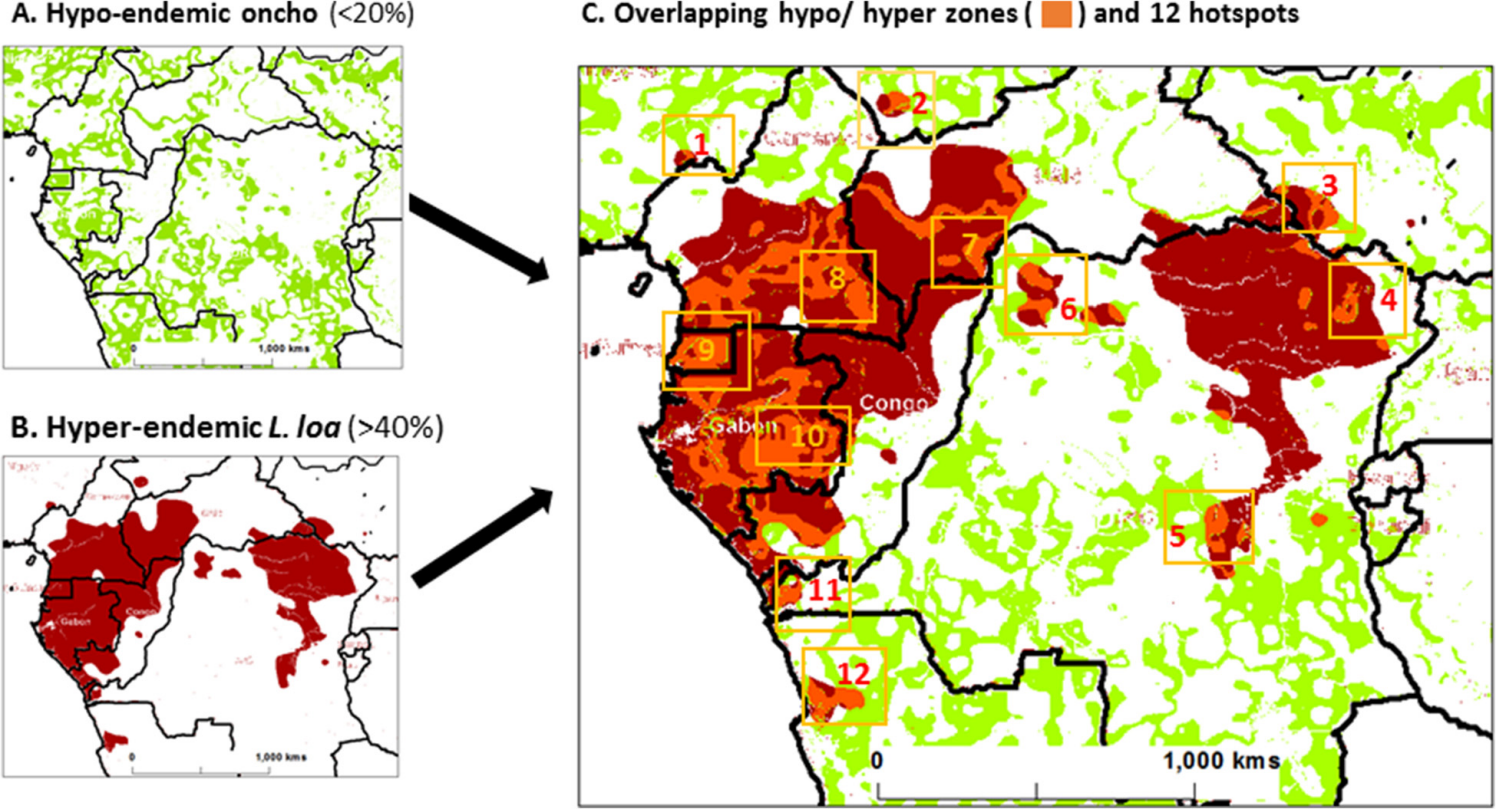
Maps of onchocerciasis and *L. loa* and overlapping hypo-and hyper-endemic zones and primary hotspots

**Fig. 2:**
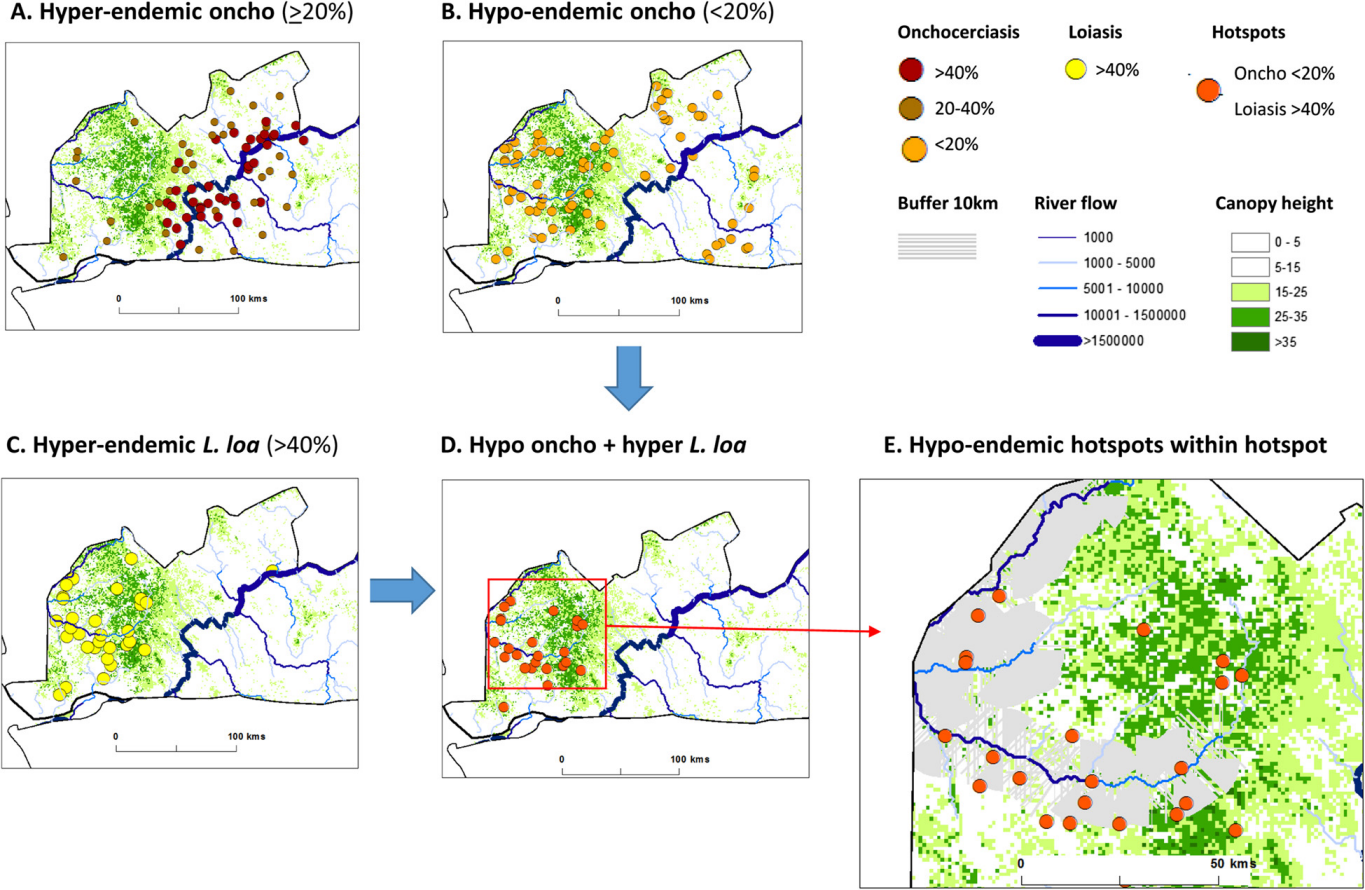
An example of micro-mapping and environmental delineation in Bas Congo, Democratic Republic of Congo

The specific steps we have undertaken to develop these new strategies for ‘*hypo-endemic hotspots’* in Central and West Africa are outlined below in bullet form in relation to Figs. 1 and 2, which were developed using GIS software ArcGIS 10 (ESRI, Redlands, CA).

#### A. Micro-stratification overlap mapping (MOM) to identify ‘*hypo-endemic hotspots*’

- The first step was to define low transmission/hypo-endemic areas of onchocerciasis. While ground-truth data are not widely available due to the lack of mapping in low risk zones, data from modelled prevalence distributions based on REMO mapping were extracted and remapped to highlight the extent of 5 % to 20 % onchocerciasis [3]. These hypo-endemic areas are illustrated in green in Fig. 1a, and show the potentially large geographical expanse of low transmission/hypo-endemic zones that need to be targeted for elimination.
- The second step was to define areas of high transmission/hyper-endemic *L. loa.* These areas were derived from data from modelled prevalence distributions from the large-scale RAPLOA map [13], which were extracted and remapped to highlight the extent of >40 % estimated loiasis prevalence. The hyper-endemic areas are illustrated in dark red in Fig. 1b, and show the large focal and unusual distribution around the Congo River basin [36].
- The third step was to overlap these stratified layers of prevalence to highlight where the hypo-endemic onchocerciasis areas geographically coincided with hyper-endemic *L. loa* areas. Figure 1c demonstrates there was irregular co-distribution, highlighted in orange, with the majority of overlapping areas in the western region of Central Africa in the five countries of Cameroon, Central Africa Republic, Congo, Equatorial Guinea (mainland) and Gabon. Smaller overlapping areas were also found in the five countries of Angola, Chad, DRC, Nigeria, and South Sudan.
- The final step was to identify selected areas which could be geographically defined as 100 to 150 km^2^ areas with overlapping hypo-endemic onchocerciasis and hyper-endemic *L. loa* and therefore defined as *‘hypo-endemic hotspots’* for further micro-mapping and environmental delineation. The 12 primary hotspots identified in Fig. 1c were found to have potentially different exposures to ivermectin as seven were in areas that coincided with CDTi areas, and may have received or be in close proximity to communities receiving ivermectin according to an APOC treatment map [45]. Completely drug naïve areas were *hotspots# 7, 8*, *9, 10 and 11* and may be considered high risk areas of SAEs if ivermectin were to be administered, and as such should be considered as pilot areas for the scale up of alternative treatment strategies.

#### B. Integrated micro-mapping to delineate environment predictors and intervention targets

- The first step was to identify the availability of prevalence distributions within a hotspot zone. *Hotspot # 11* in the Bas Congo region of DRC was used an example as onchocerciasis and loiasis prevalence data were available from an integrated micro-mapping activity, with hyper and hypo areas of both diseases across 144 sites and a reported history of SAEs in hyper-endemic areas [12, 46].
- The second step was to determine the different prevalence patterns of onchocerciasis in relation to the river systems available from remotely sensed HydroSHEDS flow accumulation data [47]. Figure 2a shows the hyper-endemic onchocerciasis areas of 20-40 % and > 40 % prevalence showing their close proximity to the Congo River itself, with the majority of sites with >40 % within 15kms of this high accumulation flow river. Figure 2b shows the wider distribution of hypo-endemic onchocerciasis, with sites further from the Congo River itself and which were found to geographically overlap to a greater degree with forested areas as represented by remotely sensed vegetation canopy height [48].
- The third step was to determine the high risk *L. loa* areas (>40 % prevalence) within the *hotspot* as this poses the greatest risk of SAEs and identifies where alternative strategies would need to be targeted at a micro-level. Figure 2c highlights the distribution of hyper-endemic *L. loa* in tropical forested areas with an average of 10 m canopy height. Figure 2d shows the specific sites with overlapping hypo-endemic onchocerciasis and hyper-endemic *L. loa* (n = 24 sites)*,* which were found to occur within an approximate 50 km^2^ area, and considered as a ‘*secondary hotspot’* (i.e. hotspot within the hotspot)
- The final step was to identify target areas within the ‘*secondary hotspot’* determined by potential key environmental proxies for low transmission such as secondary rivers and tributaries with lower flow accumulations. Figure 2e shows the 10 km buffer zone (in grey) around the rivers with lower flow accumulations and highlights that the majority of hypo/hyper sites (71 %; 17/24 sites) were within this defined riverine buffer area.

## Summary

Here we demonstrate that the combination of micro- overlapping and integrated eco-epidemiological mapping can be used to identify and delineate ‘*hypo-endemic hotspots’*of onchocerciasis where they may be at significant risk of SAEs due to the high prevalence of *L. loa*, if ivermectin is used for elimination. These areas were hitherto not regarded as being in need of treatment as it was considered that onchocerciasis was not a significant public health problem. With the emphasis on elimination of the disease from Africa by 2025, it is clear that at least a proportion of low transmission zones require some form of treatment. The previously used mapping approaches of REA, REMO and RAPLOA to identify high risk onchocerciasis and *L. loa* areas are not practical to use across the entire low transmission zones, due to the high financial costs, and the time (months to years) required to conduct surveys in remote and inaccessible areas of Central and West Africa.

We advocate that our two strategies when used together in combination with the standard rapid REA, REMO and RAPLOA methods will not only reduce the time (weeks to months) required to identify these high risk areas, but also prioritize these *hotspots* as zones where increased vigilance will be required and where resources will need to be put in place by implementing partners to ensure any SAEs are provided for adequately. While there are some limitations with finding suitably qualified experts, and the use of modelled data and remote sensing imagery e.g. different scales, resolution and some high costs of images (much less than the costs of field surveys), we consider that this approach is an important first step to understand the scope of the problem, and is a novel and practical way to at least initiate activities and pilot implementation in selected ‘*hypo-endemic hotspots’*.

## Additional file

Additional file 1:
**Multilingual abstracts in the six official working languages of the United Nations.** (PDF 282 kb)

## Abbreviations

APOC: African Programme for Onchocerciasis Control
DRC: Democratic Republic of Congo
CDTi: Community-directed treatment with ivermectin
LF: Lymphatic filariasis
Mf: Microfilaria
MOM: Micro-stratification overlap mapping
OEPA: Onchocerciasis Elimination Programme of the Americas
RAPLOA: Rapid assessment procedure of loiasis
REA: Rapid epidemiological assessment
REMO: Rapid epidemiological mapping of onchocerciasis
SAEs: Severe adverse events
MDA: Mass drug administration
NTD: Neglected tropical diseases
