# Supplementary material for: Hypo-endemic onchocerciasis hotspots: defining areas of high risk through micro-mapping and environmental delineation
Source: Infect Dis Poverty. 2015 Aug 16;4:36. doi: 10.1186/s40249-015-0069-6 (PMC4537576; doi:10.1186/s40249-015-0069-6)
Supplement: Additional file 1: — Multilingual abstracts in the six official working languages of the United Nations. (PDF 282 kb) [file 40249_2015_69_MOESM1_ESM.pdf]

Translation of the abstract into the six official working languages of the United Nations

بؤر الأنتشار العالى فى الأماكن الأقل وبائية بداء كلابية الذنب: تحديد المناطق الأكثر خطورة بإستخدام الخرائط الدقيقة والتخطيط البيئي

لويس أ. كيلى-هوب، توماس ر. يوناسش، ميشيل س. ستانتون وديفيد هـ. مولينو

الملخص

الخلفية: داء كلابية الذنب المعرف أيضا بالعمى النهري والذى ينتج عن الإصابة بطفيلى كلابية الذنب المتلوية، وينتقل عن طريق حشرات نهرية من جنس السيميوليوم (الذباب الأسود) تم التخطيط للقضاء عليه في أفريقيا حيث تحول البرنامج الأفريقي من مرحلة مكافحة الداء بالعلاج الدوائى على نطاق جماهيرى بعقار الميكتيزان® (الإيفرمكتين) فى الاماكن المتوسطة والشديدة الوبائية إلى مرحلة القضاء عليه نهائيا فى جميع الأماكن الموبوءة والذى يتطلب العديد من الاحتياطات، وتعتبر التحديات التى تواجه القضاء النهائى على داء كلابية الذنب فى الاماكن الاقل وبائية او التى بها نسبة انتقال اقل هى تحديات مضاعفة نظرا لوجود العديد من الاماكن النائية التى بها بؤر إنتقال أقل وغير محددة نسبيا بالإضافة إلى أن المعالجة بالإيفرمكتين يسبب آثار جانبية خطيرة في الأشخاص الذين يعانون من وجود نسبة عالية من طفيلى اللوا لوا فى الدم، طفيلي الفيلاريا المنتشر على نطاق واسع في وسط وغرب أفريقيا، والمسئول عن داء دودة العين الاستوائية وينتقل عن طريق ذباب الخيل من جنس كريسوبس.

المناقشة: ومن ثم فإننا نقترح منهجا حديثا لرسم خرائط داء كلابية الذنب بإستخدام الأقمار الصناعية والاستشعار عن بعد ونماذج البيانات البيئية جنبا الى جنب مع المسوحات الميدانية للمساعدة في حل المشاكل التى تعوق التوسع فى نطاق القضاء على داء كلابية الذنب فى الاماكن الموبوءة باللوا لوا حيث بينا أن الخرائط الدقيقة متداخلة الطبقات للخرائط المتاحة لإنتشار دائى كلابية الذنب واللوائيات يمكن استخدامها لتحديد إثنتى عشرة منطقة عالية الخطورة يتداخل فيها إنخفاض كلابية الذنب المتلوية مع إرتفاع اللوا لوا والتى تم تعريفها بالبؤر الجغرافية ذات الانتشار العالى فى المناطق الاقل وبائية كما أكدنا على أن إستخدام الخرائط الدقيقة المتكاملة لبيانات انتشار كلابية الذنب وإستخدام البيانات البيئية فى تحديد عوامل الخطر المرتبطة بأماكن معيشة ذبابتى السيميوليوم والكريسوبس فى الغابات والمجارى النهرية من الممكن ان يساعد على تحديد المناطق التى تحتاج للتدخل أو المناطق ذات الخطورة الثانوية داخل مناطق الانتشار العالى لداء اللوائيات ليساعد على تجنب الآثار الجانبية الخطيرة المتعلقة بالعلاج بعقار الإيفرمكتين.

الخلاصة: أمثلة هذه الخرائط المقترحة توضح قيمة الجمع بين بيانات إنتشار المرض والحشرات الناقلة له و البيانات البيئية لوضع خرائط لتنفيذ المخططات والاستراتيجيات المستهدفة وهذا أمر بالغ الأهمية حيث أن الخرائط الجيدة ربما تخفض تكلفة المكافحة وتقلل المخاطر المرتبطة باللوا لوا، ولا سيما إذا كان هناك مساحات واسعة من المناطق الأقل وبائية والتى قد تتطلب العلاج بعقار ايفرمكتين أو تتطلب استخدام إستراتيجيات بديلة ونستخلص من ذلك أن إستخدام الطرق الحديثة الفعالة من حيث التكلفة يعد أمرا ضروريا إذا أردنا القضاء على إنتقال كلابية الذنب المتلوية في أفريقيا بطريقة أمنة وذات كفاءة بحلول عام 2025.

Translated from English version into Arabic by Mohamed R. Habib, through

TRANSLATORS WITHOUT BORDERS

次流行盘尾丝虫病热点：通过微测与环境描绘界定高风险区域

Louise A. Kelly-Hope, Thomas R. Unnasch, Michelle C. Stanton, David H. Molyneux

摘要：

引言：非洲旨在消除由旋盘尾丝虫（*Onchocerca volvulus*）引起的盘尾丝虫病（即河盲症），其通过蚋（*Simulium* spp.）传播。防治策略发生了明显的变化，从利用群体服用伊维菌素控制中度和重度流行区转变为在所有流行区采用一系列干预措施用于消除。而在低度或超低流行区消除该病的挑战是成倍的。首先，低流行区存

在大量疫点相对不明确的偏远地区。其次，伊维菌素会增加罗阿丝虫（*Loa loa*）高虫血症患者的重症不良反应的风险。罗阿丝虫广泛分布于中非和西非地区，可引起热带眼虫病，通过虻传播。
讨论：建议利用遥感技术和建模环境数据的新型绘图方法，结合快速现场调查，解决在罗阿丝虫共流行区域确定开展消除盘尾丝虫病活动的问题。首先，对可利用的盘尾丝虫病和罗阿丝虫病地图的微分层重叠映射（micro-stratification overlap mapping, MOM)可以鉴定 12 个关键高风险区域，那些地区是盘尾丝虫病传播低但罗阿丝虫病传播高的重叠区域，将其定义为“次流行热点”。其次，综合患病率数据的微测和环境数据描述与蚋和虻生境相关的河流和森林风险因素有利于进一步明确干预措施的目标区域（如热点的二级热点），从而有助于避免重症不良反应。
小结：以上绘图案例证明了联合患病率、昆虫学和生态学信息为计划实施方案和制定策略制图的价值。因为优秀的绘图可以降低成本，同时降低罗阿丝虫相关的风险，尤其是那些广泛的低流行区可能还需要伊维菌素的治疗或使用其他应对措施。如果非洲到 2025 年要以高效安全的方式消除盘尾丝虫病，那么必须应用一些新型的成本效益好的方法。

Translated from English version into Chinese by Yin Jian-hai, through

CHINESE CENTER FOR DISEASE CONTROL AND PREVENTION
NATIONAL INSTITUTE OF PARASITIC DISEASES

Points chauds hypoendémiques d’onchocercose : définition des zones à haut risque par microcartographie et délimitation écologique

Louise A. Kelly-Hope, Thomas R. Unnasch, Michelle C. Stanton, David H. Molyneux

Résumé

Contexte : L’onchocercose (cécité des rivières), causée par le parasite *Onchocerca volvulus* et transmise par les simulies riveraines *Simulium* spp., est une maladie que l’on cherche à éradiquer en Afrique. La stratégie est en train d’évoluer nettement du « contrôle » des zones méso- et hyperendémiques par administration massive de Mectizan® (ivermectine) à « l’élimination » de la maladie dans toutes les zones d’endémie, qui peut nécessiter un arsenal d’interventions diverses. Les principales difficultés de l’éradication dans les zones hypoendémiques de faible transmission sont doubles : d’une part, il existe de vastes régions isolées dans lesquelles l’aspect focal des zones de faible transmission est relativement méconnu. D’autre part, le traitement par l’ivermectine crée un risque important d’effets indésirables graves pour les sujets présentant également une charge parasitaire importante de *Loa loa*, une filaire parasite très répandue en Afrique Centrale et de l’Ouest, transmise par les mouches du genre *Chrysops* et cause de la filariose oculaire.
Discussion : En conséquence, nous proposons des approches nouvelles de la cartographie, utilisant, d’une part, des données d’environnement télédétectées par satellite et modélisées et, d’autre part, des relevés rapides sur le terrain, afin de tenter de résoudre les problèmes de ciblage des activités étendues d’éradication de l’onchocercose dans les zones de *Loa loa* coendémique. Premièrement, nous démontrons que la micro-stratification et de juxtaposition des cartes de prévalence de l’onchocercose et de la filariose peut être utilisée pour identifier douze zones clés à haut risque, où la transmission d’*O. volvulus* et de *L. loa* se superpose et que nous appelons « points chauds hypoendémiques ». Deuxièmement, nous montrons que la microcartographie intégrée des données de prévalence et

l'utilisation des données écologiques pour délimiter les facteurs de risque riverains et forestiers associés aux habitats des vecteurs *Simulium* spp. et *Chrysops* spp. peuvent aider à définir des zones cibles d'intervention, autrement dit des points chauds dans les points chauds, afin d'éviter les risques d'effets secondaires graves des médicaments.

Conclusions : Ces exemples de cartographie montrent l'intérêt qu'il peut y avoir à regrouper les informations de prévalence, entomologiques et écologiques pour élaborer des cartes en vue d'une réalisation planifiée et de stratégies ciblées. Cet intérêt est crucial car une meilleure cartographie a le pouvoir de réduire le coût ainsi que les risques associés au traitement de *L. loa*, notamment lorsqu'il existe de vastes zones de faible endémicité susceptibles de nécessiter un traitement par l'ivermectine ou l'adoption d'une autre stratégie. De nouvelles approches économiques sont nécessaires si l'on veut éliminer la transmission d'*O. volvulus* de manière efficace et sûre d'ici le terme fixé de 2025.

Translated from English version into French by Suzanne Assenat, through

TRANSLATORS
WITHOUT BORDERS

Очаги с гипоэндемичным уровнем инвазированности онхоцеркозом: определение районов высокой степени риска методом микрокартографирования и воспроизведения деталей местности на карте

Louise A. Kelly-Hope, Thomas R. Unnasch, Michelle C. Stanton, David H. Molyneux

Резюме

Краткая информация: Онхоцеркоз (речная слепота), вызываемый паразитами *Onchocerca volvulus* и передаваемый при укусах обитающих по берегам рек мошек рода *Simulium spp.* является мишенью для элиминации в Африке. Речь идет о существенном изменении в стратегии: от «контроля» мезо- и гиперэндемичных районов с помощью массового лечения (MDA) препаратом Mectizan® (ивермектин) к «элиминации» во всех эндемичных районах, где только могут потребоваться подобные мероприятия. Наиболее значительные проблемы элиминации в районах с низкой интенсивностью передачи или гипоэндемичных районах имеют двоякую подоплеку. Во-первых, есть обширные отдаленные районы, где очаги с низкой интенсивностью передачи остаются относительно неопределенным. Во-вторых, лечение ивермектином увеличивает риск серьезных нежелательных явлений (СНЯ) у лиц с высоким уровнем паразитемии, вызванной филяриями *Loa loa,* широко распространенными в Центральной и Западной Африке, которые являются возбудителями лоаоза и передаются через укусы слепней рода *Chrysops spp.*

Рассмотрение: В связи с этим мы предлагаем новые подходы картографирования с использованием спутника дистанционного зондирования, а смоделированные данные о местности должны использоваться в комбинации с быстрыми полевыми работами в целях решения проблем, связанных с расширением деятельности по элиминации онхоцеркоза в эндемичных районах по *L. loa.* Во-первых, мы показываем, что микростратификационное дублированное картографирование (МОМ) на основе имеющихся карт по пораженности онхоцеркозом и лоаозом может использоваться для идентификации 12 ключевых районов

повышенного риска с низкой интенсивностью передачи *O. volvulus* и высокой — L. loa, которые мы определяем как «гипоэндемичные очаги». Во-вторых, мы показываем, что микрокартографирование с использованием данных о пораженности в комплексе с воспроизведением на карте данных о речных и лесных факторах риска, связанных со средой обитания переносчиков *Simulium spp.* и *Chrysops spp.*, может в дальнейшем помочь в определении целевых районов для вмешательства, т.е. вторичных очагов в очагах, тем самым способствуя уменьшению рисков СНЯ.

Выводы: Данные примеры картографирования показывают смысл сведения воедино показателей пораженности, энтомологической и экологической информации при составлении карт для осуществления плановых мероприятий и целевых стратегий. Решающее значение имеет тот факт, что лучшее качество картографирования может снизить затраты и риски, связанные с *L. loa,* особенно если существуют обширные районы с низким уровнем эндемичности, где может потребоваться лечение ивермектином или применение альтернативных стратегий. Если, согласно цели 2025 г., элиминация *O. volvulus* в Африке должна достигаться эффективными и безопасными путями, то для этого необходимы новые низкозатратные подходы.

Translated from English version into Russian by Anna Romanenko, through

TRANSLATORS WITHOUT BORDERS

Zonas de conflicto para la oncocercosis hipo-endémica: cómo definir las áreas de alto riesgo a través de micro-mapeo y delineación

Louise A. Kelly-Hope, Thomas R. Unnasch, Michelle C. Stanton, David H. Molyneux

Resumen

Antecedentes: En África se ha puesto como meta la eliminación de la oncocercosis (ceguera de los ríos) que produce el parásito *Onchocercavolvulus* y transmite la *Simulium*spp. (moscas negras) que habita en los ríos. Este es un cambio significativo en la estrategia de "control" de áreas meso e hiper-endémicas mediante la administración masiva de medicamentos (MDA) con Mectizan® (ivermectin), a la "eliminación" en todas las áreas endémicas donde es posible que se requiera de una serie de intervenciones. Los desafíos más significativos de la eliminación en áreas de baja transmisión o hipo-endémicas tienen dos aspectos. En primer lugar, existen vastas áreas remotas donde la focalidad de la baja transmisión es relativamente indefinida. En segundo lugar, el tratamiento con ivermectin incrementa el riesgo de eventos adversos serios en individuos con parasitemias elevadas de *Loa loa*, un parásito filarial ampliamente diseminado en África Central y Occidental, que produce el gusano del ojo africano y que se transmite por la*Chrysops*spp. (moscas del venado).

Discusión: Por lo tanto proponemos un abordaje de mapeo totalmente nuevo mediante el uso de satélites con sensor remoto e información de modelado ambiental a usarse en combinación con encuestas rápidas de campo para ayudar a resolver los problemas del abordaje de la expansión de las actividades de eliminación de la oncocercosis en áreas donde la *Loa loa* es co-endémica. Primero, demostramos que la micro-estratificación del mapeo con

solapamiento de mapas disponibles de prevalencia de oncocercosis y loiasis puede ser utilizada para identificar 12 áreas fundamentales de alto riesgo, donde una baja transmisión de *O. volvulus* y una alta transmisión de *L. loa* se superponen, lo que definimos como "zonas de conflicto hipo-endémicas". Segundo, mostramos que el micromapeo integrado de la información de prevalencia y el uso de información ambiental para delinear los factores de riesgo fluviales y forestales asociados con los hábitats vectoriales de *Stimuliumspp*. y*Chrysopsspp*. pueden ayudar incluso más aún a definir las áreas objetivo de intervención, es decir, zonas de conflicto secundarias dentro de las zonas de conflicto, para ayudar a evitar el riesgo de eventos adversos serios.

Resumen: Estos ejemplos de mapeo demuestran el valor de la combinación de la información de prevalencia, entomológica y ecológica para desarrollar mapas y para la implementación planificada y estrategias dirigidas. Esto es de importancia crítica ya que un mejor mapeo puede disminuir los costos y reducir los riesgos asociados a la *L. loa*, especialmente si existen vastas áreas de bajoendemismo que puedan llegar a requerir tratamiento con ivermectin o estrategias alternativas. Se necesita de abordajes nuevos y económicos para lograr la eliminación eficiente y segura de la transmisión de *O. volvulus* en Áfricade acuerdo a la meta establecida para el año 2025.

Translated from English version into Spanish by Maria Alejandra Aguada, through

TRANSLATORS
WITHOUT BORDERS
